# Validity and reliability of the Iranian version of the Pediatric Quality of Life Inventory™ 4.0 (PedsQL™) Generic Core Scales in children

**DOI:** 10.1186/1477-7525-10-3

**Published:** 2012-01-05

**Authors:** Parisa Amiri, Ghazaleh Eslamian, Parvin Mirmiran, Niloofar Shiva, Mohammad Asghari Jafarabadi, Fereidoun Azizi

**Affiliations:** 1 Obesity Research Center, Research Institute for Endocrine Sciences, Shahid Beheshti University of Medical Sciences, Tehran, Iran; 2 Student Research Committee, Shahid Beheshti University of Medical Sciences, Tehran, Iran; 3 Endocrine Research Center, Research Institute for Endocrine Sciences, Shahid Beheshti University of Medical Sciences, Tehran, Iran; 4 National Public Health Management Center (NPMC) and Department of Statistics and Epidemiology, Faculty of Health and Nutrition, Tabriz University of Medical Sciences, Tabriz, Iran

**Keywords:** Health-related quality of life, PedsQL™, Iran, Children

## Abstract

**Background:**

This study aimed to investigate the reliability and validity of the Iranian version of the Pediatric Quality of Life Inventory™ 4.0 (PedsQL™ 4.0) Generic Core Scales in children.

**Methods:**

A standard forward and backward translation procedure was used to translate the US English version of the PedsQL™ 4.0 Generic Core Scales for children into the Iranian language (Persian). The Iranian version of the PedsQL™ 4.0 Generic Core Scales was completed by 503 healthy and 22 chronically ill children aged 8-12 years and their parents. The reliability was evaluated using internal consistency. Known-groups discriminant comparisons were made, and exploratory factor analysis (EFA) and confirmatory factor analysis (CFA) were conducted.

**Results:**

The internal consistency, as measured by Cronbach's alpha coefficients, exceeded the minimum reliability standard of 0.70. All monotrait-multimethod correlations were higher than multitrait-multimethod correlations. The intraclass correlation coefficients (ICC) between the children self-report and parent proxy-reports showed moderate to high agreement. Exploratory factor analysis extracted six factors from the PedsQL™ 4.0 for both self and proxy reports, accounting for 47.9% and 54.8% of total variance, respectively. The results of the confirmatory factor analysis for 6-factor models for both self-report and proxy-report indicated acceptable fit for the proposed models. Regarding health status, as hypothesized from previous studies, healthy children reported significantly higher health-related quality of life than those with chronic illnesses.

**Conclusions:**

The findings support the initial reliability and validity of the Iranian version of the PedsQL™ 4.0 as a generic instrument to measure health-related quality of life of children in Iran.

## Background

Health-related quality of life (HRQOL) measures are increasingly being used in an effort to continually improve the quality of the healthcare for pediatric patient health in clinical trials [1], population health [2], clinical improvement [3], and among purchasers of health care services [4]. Today, most descriptions of HRQOL refer to it as a multidimensional construct [5] that focuses on individuals' subjective evaluation of their physical, psychological (including emotional and cognitive), and social health dimensions delineated by the World Health Organization (WHO) [6].

There are numerous of well-developed generic and disease specific HRQOL measures for children and adolescents [7]. To integrate the merits of generic and disease-specific instruments for children and adolescents, aged 2-18 years old, the Pediatric Quality of Life Inventory™ (PedsQL™) was designed and developed in the US [8]. The PedsQL™ 4.0 Generic Core Scales and disease-specific questionnaires have resulted from iterative process and are applicable for healthy schools [9] and community populations [10], as well as pediatric populations with acute [11] and chronic health conditions, such as cancer, cerebral palsy, diabetes, rheumatologic diseases, and end-stage renal disease [12-16]. The PedsQL™ 4.0 Generic Core Scales include child self-report and parent proxy-report forms and can be completed easily [10]; the US English version of the PedsQL has been linguistically validated in many non-English-speaking countries [17-20].

Childhood is the crucial phase for overall development, including physical, psychological, and social development, throughout an individual's lifespan [21]. Health-related quality of life assessment for children may be useful in targeting interventions and directing resources to individuals and communities. Moreover, as cultural differences may exist in the assessment of HRQOL, nation-specific information is required to enable national and international evaluation and benchmarking.

We have previously reported the initial reliability and validity of the Iranian version of the PedsQL™ 4.0 as a generic instrument to measure HRQOL of a general population of Iranian adolescents, aged 13-18 years [22]. Another study conducted on attention deficit/hyperactivity disorders in Iranian children and adolescents, aged 8-17 years, reported the psychometric properties of the PedsQL™ [23]; given the limited sample size of the study mentioned and considering that the PedsQL™ originally has two separate scales for children (8-12 years old) and adolescents (13-18 years old) that makes a single statistical analysis and conclusions difficult and vague, the current study, aimed to investigate reliability and validity of the Iranian version of the PedsQL™ 4.0 generic core scale among a large number of Iranian children, aged 8-12 years. Based on previous studies from international back translations of the PedsQL™ 4.0, we hypothesized that the PedsQL™ 4.0 could also demonstrate satisfactory psychometric properties in Iranian children and would hence differentiate HRQOL between a healthy pediatric population and one with chronic health conditions.

## Methods

### Participants

Participants were 649 children, aged 8-12 years, who were recruited from primary and secondary schools in Tehran, and their parents. The inclusion criteria were obtaining of agreement from both the children and their parents, who were required to give their written informed consent to participate. Overall 525 children and their parents agreed to take part in the study, giving a response rate of 80%. No significant differences were observed in age, gender, health status and their residential area between participants and non-participants. Three hundred and thirty-two (63.2%) of the children were girls and 503 (95.8%) were healthy (did not suffer from any chronic health condition). All questionnaires were completed anonymously. Twenty-two children, aged 8-12 years, were recruited from university hospitals with identified chronic health conditions including asthma (n = 3), renal failure (n = 8), and cancer (n = 11). The study protocol was approved by the ethics committee of the Obesity Research Center, Research Institute for Endocrine Sciences, Shahid Beheshti University of Medical Sciences.

### Measures

#### PedsQL™ 4.0 Generic Core Scales

The 23-item PedsQL™ 4.0 Generic Core Scales is a self-administered questionnaire that includes child self-reports and parent proxy-reports, which encompass the following subscales: Physical Functioning (8 items), Emotional Functioning (5 items), Social Functioning (5 items) and School Functioning (5 items). A 5-point Likert response scale ranging from 0 (never a problem) to 4 (almost always a problem) is used across child self-reports for ages 8-18 and parent proxy-reports. According to the manual of the instrument, if more than 50% of the items in the scale are missing, the scale score is not computed. The total scale scores for both child self-report and parent proxy-report were also calculated [8,10]. In addition to the PedsQL™ 4.0 questionnaires, all families were required to complete a family information form on socio-demographic and child health characteristics.

### Procedure

#### Translation

The Iranian (Persian) translation and linguistic validation of the PedsQL™ 4.0 questionnaire followed recommended guidelines [24]. This process included using two translators, who are a health educator and a clinical psychologist independently. To produce a conceptual equivalence of the translation to the original English questionnaire, both translators discussed any disparities and agreed on a single version. The backward translation of the first reconciled forward version of the PedsQL™ 4.0 questionnaire to the original U.S. English version was performed by two local professional translators who were not associated with the first translation phase with experience of living in English-speaking countries. In a pre-test, the PedsQL™ was given to 50 children and their parents to ensure confidence in the linguistic and conceptual equivalence of the translations. Cognitive interviewing technique was also used to find and correct errors introduced through the translation process. The relevant changes in the translation process were reviewed for conceptual equivalence and authorized by the principal developer of the PedsQL™ (Dr. Varni).

### Data collection

Participants were selected from four primary and secondary schools, located in the north of Tehran. All the schools were selected using stratified random sampling methods, considering level of education and gender. Participants from two schools from each sex and level were recruited for the study. Trained research personnel visited each classroom and distributed a package including a written consent form, cover letter, family information form and the PedsQL™ for the parents to fill out at home. The cover letter explained the study and guaranteed the confidentiality of data, assuring that even the school staff would not see the information. The participants could contact the researchers to get further information and guidance. After the research team had collected the questionnaires which were returned to the school, project staff revisited each class and administered the PedsQL™ 4.0 to those children, whose parents had completed the questionnaires at home and signed consent forms. Subjects who suffered chronic health conditions were recruited from two university hospitals. After receiving informed consents from parents, the questionnaires were completed by children and their parents separately. Trained research personnel assisted participants in completing the questionnaires.

### Statistical analysis

The total score of each scale was computed by summing up items related to the scale and used in the analysis. The data were presented as "Mean ± SD" for the variables. To determine whether univariate normality exists, we examined the distribution of each observed variable for skewness and kurtosis. For the skewness index, absolute values above than 3.0 are extreme [25]. Absolute values higher than 10.0 for the kurtosis index, suggest a problem, [26].

The feasibility of the Iranian version of the PedsQL™ 4.0 was determined based on the percentage of missing values for each item. Ceiling and floor effects were evaluated based on percentage of scores at the extremes of the scaling range [27]. Floor or ceiling effects are considered to be present if more than 15% of respondents achieve the lowest or highest possible score, respectively [28]. Internal consistency (to test reliability) was assessed by calculating Cronbach's alpha (α) coefficient [29]. Alpha coefficients equal to or greater than .70 were considered satisfactory. We computed the intraclass correlation coefficient (ICC) to evaluate child self-report and parent proxy-report agreement on the PedsQL™ 4.0 subscales. ICCs ≤ 0.4 were considered poor to fair agreement; 0.41-0.60 moderate agreement; 0.61-0.80 good agreement and > 0.80 excellent agreement [30]. The multitrait-multimethod was used to compute parent-child Pearson intercorrelations between and among PedsQL™ 4.0 subscales. Correlations are designated as small (0.10-0.29), medium (0.30-0.49), and large (≥ 0.50) [31]. Factor structure of the PedsQL™ 4.0 was extracted using exploratory factor analysis (EFA), utilizing principal component analysis and varimax rotation. To assess how well the EFA extracted model fits observed data, we conducted confirmatory factor analysis (CFA), using the method of weighted least squares for estimation. Asymptomatic covariance matrix was considered a weighted matrix. Input matrix was covariance matrix of data. Fit indices and reasonable values of these indices for CFA were considered as χ^2^/df < 5, Root Mean Square Error of Approximation (RMSEA) < 0.08 and also, Comparative Fit Index (CFI), Goodness of Fit Index (GFI), Adjusted Goodness of Fit Index (AGFI) > 0.9 [32]. Given previous PedsQL™ CFI findings, 5- and 6-factor models were tested [33-35]. Construct validity was tested performing the known-groups method which compares scale scores across groups known to differ in the health construct being surveyed. We hypothesized that healthy children would report higher scores than children with a chronic health condition. Student's *t *test was performed to determine gender differences between parent proxy-report and child self-report. Statistical analysis was performed using SPSS 15.0 (SPSS Inc., Chicago, IL) and LISREL 8.80 (Scientific Software International Inc., 2007). *P-*Values less than 0.05 were considered significant.

## Results

Parent proxy-reports were completed by 397 (76.2%) mothers, 114 (21.9%) fathers and 10 (1.9%) by other caregivers such as grandparents. Missing responses for items were rare and ranged from 0.0 to 1.9 percent for both the child self-report and parent proxy-report. In the chronic health condition sample, missing responses ranged from 0.0 to 4.5 percent.

The internal consistency of the scale as measured by Cronbach's alpha coefficients showed that all child self- and parent proxy-report subscales of the PedsQL™ 4.0 exceeded the minimum reliability standard of 0.70, except for emotional functioning for children and school functioning for both respondents. The total scale scores internal consistency alphas were 0.84 and 0.88 for child self-report and parent proxy-report, respectively. No floor effects were observed while ceiling effects detected ranged from 2.9%, for self-report total score, to 37.7% for self-report social functioning (Table 1).

**Table 1:** Means, standard deviations, percent floor and ceiling effects, and Cronbach's α for the Iranian version of PedsQL™ 4.0 generic core scales (n = 525)

	Mean	SD	Skewness	Kurtosis	Percent floor	Percent ceiling	Cronbach's α
**Child self-report**							
Total score	83.99	11.90	-1.26	1.76	0	2.9	0.84
Physical functioning	85.29	13.95	-1.44	4.32	0	5.5	0.70
Emotional functioning	80.55	16.75	-0.92	0.54	0	5.9	0.64
Social functioning	86.88	16.01	-1.44	3.18	0	37.7	0.70
School functioning	83.26	14.88	-1.36	3.35	0.2	19.6	0.60
**Parent proxy-report**							
Total score	76.75	13.45	-0.31	-0.15	0	3.0	0.88
Physical functioning	77.52	18.48	-0.74	0.47	0	17.7	0.83
Emotional functioning	70.95	17.37	-0.22	-0.74	0	5.7	0.70
Social functioning	80.77	17.89	-0.83	0.10	0	25.3	0.74
School functioning	77.76	15.77	-0.47	0.19	0.2	13.9	0.63

Floor or ceiling effects are considered to be present if more than 15% of respondents achieve the lowest or highest possible score, respectively.

All monotrait-multimethod correlations demonstrated a moderate relation between child self-report subscales and parent proxy-report subscales, ranging from 0.37 to 0.43 (Table 2). All multitrait-multimethod correlations were lower than the monotrait-multimethod correlations. The average convergent correlation was 0.40 and the average off-diagonal correlation was 0.30. The intraclass correlation coefficients (ICC) were moderate to high for all scales, indicating good agreement between child and parent reports except for the total scale scores, which showed a reasonable agreement (ICC = 0.67, 95% CI: 0.61-0.72). Lowest agreement was detected for the social functioning scale (ICC = 0.55, 95% CI: 0.46-0.62).

**Table 2:** Intercorrelations of children self-report and parent proxy-report for the Iranian version of PedsQL™ 4.0 Generic Core Scales by the Multitrait-Multimethod Matrix (n = 525)

	Child self-report				Parent proxy-report		
	
	Physical	Emotional	Social	School	Physical	Emotional	Social
**Child self-report**							
Physical functioning							
Emotional functioning	**0.42**						
Social functioning	**0.52**	**0.39**					
School functioning	**0.50**	**0.43**	**0.51**				
**Parent proxy-report**							
Physical functioning	**0.40**	*0.25*	*0.31*	*0.29*			
Emotional functioning	*0.29*	**0.39**	*0.28*	*0.33*	**0.47**		
Social functioning	*0.28*	*0.23*	**0.37**	*0.28*	**0.53**	**0.42**	
School functioning	*0.20*	*0.25*	*0.23*	**0.43**	**0.46**	**0.45**	**0.42**

All correlations were significant at *P *< 0.001

Multitrait-monomethod correlations are in bold; Monotrait-multimethod correlations are underlined; Multitrait-multimethod correlations are italicized.

Exploratory factor analysis with varimax rotation extracted six factors from the PedsQL™ 4.0 for both self- and proxy- reports, accounting for 47.9 and 54.8% of total variance, respectively. Kaser-Meier-Olkin (KMO) values of 0.71 and 0.74 respectively for children and parents EFA and P < 0.001 of Bartelet test of sphericity for both children and parents confirmed the adequacy of the factor model as well. The two physical functioning items measuring pain and fatigue were split into a different factor for both the child self-reports and the parent proxy-reports. In the parent-report, the first factor consisted of items 1 to 6 and the second factor consisted of items 7 and 8; however in the children report, the first factor consisted of items 1 to 4 and the second factor consisted of items 5 to 8. Similarly, the school functioning scale was split into two factors; in the parent and child reports, the first factor consisted of items 1 to 3 and second factor consisted of items 4 and 5 (Table 3). The results of the CFA for 5- and 6-factor models for self- and parent proxy-reports indicated a more acceptable fit for the 6-factor model. In addition all parameters relating to the factors and indicators were statistically significant (All P < 0.05) (Table 4).

**Table 3:** Factor analysis results for child self-report (left) and the parent proxy-report (right) for the Iranian version of the PedsQL ™ 4.0 Generic Core Scales

	Factor 1		Factor 2		Factor 3		Factor 4		Factor 5		Factor 6	
**Physical functioning**												
1. It is hard for me to walk more than one block	**0.78**	**0.71**	0.01	0.30								
2. It is hard for me to run	**0.73**	**0.73**	0.22	0.38								
3. It is hard for me to do sports activities or exercise	**0.56**	**0.78**	0.39	0.26								
4. It is hard for me to lift something heavy	**0.53**	**0.39**	0.05	0.52								
5. It is hard for me to take a bath or shower by myself	-0.07	**0.77**	**0.77**	0.02								
6. It is hard for me to do chores around the house	0.14	**0.71**	**0.57**	0.14								
7. I hurt or ache	0.16	0.15	**0.49**	**0.78**								
8. I have low energy	0.46	0.12	**0.51**	**0.81**								
**Emotional functioning**												
1. I feel afraid or scared					**0.68**	**0.70**						
2. I feel sad or blue					**0.61**	**0.76**						
3. I feel angry					**0.66**	**0.68**						
4. I have trouble sleeping					**0.61**	**0.59**						
5. I worry about what will happen to me					**0.64**	**0.61**						
**Social functioning**												
1. I have trouble getting along with other kids							**0.69**	**0.76**				
2. Other kids do not want to be my friend							**0.73**	**0.80**				
3. Other kids tease me							**0.59**	**0.66**				
4. I cannot do things other kids my age can do							**0.68**	**0.72**				
5. It is hard to keep up with my peers							**0.63**	**0.55**				
**School functioning**												
1. It is hard to pay attention in class									**0.80**	**0.84**	0.00	0.00
2. I forget things									**0.71**	**0.72**	0.04	0.12
3. I have trouble keeping up with my schoolwork									**0.62**	**0.75**	0.28	0.14
4. I miss school because of not feeling well									0.01	0.10	**0.87**	**0.84**
5. I miss school to go to the doctor or hospital									0.18	0.09	**0.81**	**0.84**

**Table 4:** Goodness of fit indices for 5- and 6-factor CFA models of parent and child reports (n = 525)

Model	x2	df	x2/df	RMSR	RMSEA (90% CI)	CFI	GFI	AGFI
**Parents-6 factor model**	685.81 *	211	3.25	0.058	0.066 (0.060; 0.071)	0.95	0.90	0.87
**Parents-5 factor model**	1261.72 *	220	5.74	0.065	0.075 (0.071; 0.079)	0.94	0.89	0.86
**Children-6 factor model**	609.74 *	214	2.85	0.052	0.059 (0.054; 0.065)	0.94	0.91	0.88
**Children-5 factor model**	1242.72 *	220	5.65	0.061	0.074 (0.070; 0.078)	0.94	0.89	0.86

CFA, confirmatory factor analysis; x2, chi-square; df, degrees of freedom; x2/df, normed chi-square; RMSR, root mean square residual; RMSEA, root mean square error of approximation; CFI, comparative fit index; GFI, goodness of fit index; AGFI, adjusted goodness of fit index

* P < 0.001

**Figure 1:**
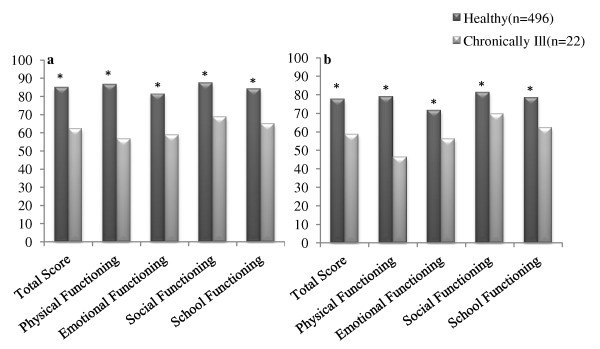
Comparison of PedsQL™ 4.0 scores between healthy and chronically ill children. a) Child Self- report, b) Parent-proxy report, *P < 0.01.

**Figure 2:**
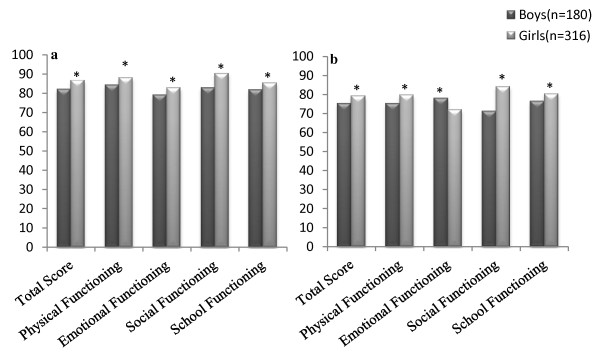
Comparison of PedsQL™ 4.0 scores between boys and girls. a) Child Self- report, b) Parent-proxy report, *P < 0.01.

There were statistically significant differences between healthy and chronically ill children for all subscales, where children with chronic health conditions reported lower scores than did healthy children (Figure 1). Also among healthy participants, a gender difference was found in all subscales and total scores for child self-report. Compared to boys, girls scored higher on the total scale score (86.7 ± 9.9 vs. 82.02 ± 11.2, *P *< 0.01), physical functioning (88.1 ± 13.1 vs. 84.2 ± 11.7, *P *< 0.001), emotional functioning (79.2 ± 16.1 vs. 82.8 ± 15.9, *P *= 0.01), social functioning (90.4 ± 14.0 vs. 82.8 ± 15.9, *P *< 0.001) and school functioning (85.5 ± 12.7 vs. 81.7 ± 15.0, *P *= 0.003) (Figure 2).

## Discussion

This study investigated the psychometric properties of the Iranian version of the PedsQL™ 4.0 Generic Core Scales in children aged 8-12 years. Our results indicate the preliminary reliability and validity of the Iranian version of PedsQL™ 4.0 as a child self-report and parent proxy-report measure of generic HRQOL in Iran.

Our study presents, the feasibility of PedsQL™ 4.0, as measured by a low percent of missing values, particularly in healthy children. Similar to previous studies, no floor effects were found in either the self-report or parent proxy versions [8,17,22,36]. Most subscales in this study indicated some ceiling effects, which support results of previous studies [18,22,36].

Cronbach's α coefficient to test reliability were acceptable (exceeded .70) for all measures and showed strong internal consistency reliability for the total scale, and most subscales including physical, emotional and social functioning in both children and parents. This satisfactory level of internal consistency is almost similar to the original version and other translated versions [8,19,37].

The multitrait-monomethod correlations were medium for child self-report and for parent proxy-report. Our results indicated that the multitrait-multimethod correlations were smaller than the monotrait-multimethod correlations, providing evidence for the validity of the instrument's dimensions. In general, there was a good agreement between children and parent reports except for the total scale scores which had also been observed in our previous study [22] and could have been due to strong parental support in Iranian families. These findings are inconsistent with the original version and most of the other translated versions [17,18,38].

Factor validity of the scales for parent proxy-reports and child self-reports was determined through factor analysis. According to our results, the 6-factor model showed much a better fit than the 5-factor model based on the good of fitness (GOF) indices from CFA. In addition based on the criteria used (Scree test) and theoretical consideration, the results of EFA on this set of data, were best fitted with a 6-factor solution. However our current EFA/CFA findings are not in agreement with the results of our previous study on the Iranian version of PedsQL Generic Core Scales in adolescents [22], or with those from another study conducted to assess the PedsQL™ Oral Health Scale in Iranian children [39] findings which both support a priori 5-factor model; the EFA/CFA findings of yet another study of a much smaller sample of Iranian children and adolescents with attention deficit/hyperactivity disorder ADHD, showed an acceptable fit of a 4-factor model [23].

In this study, for child self-report, the items related to physical functioning were loaded in two separate factors each containing 4 items. Item 5 to 8 were loaded to an independent factor (factor 2). All items related to emotional functioning and social functioning had a clear factor loading. For parent proxy-report, two items related to physical functioning (items 7 and 8) were loaded to other factors. One possible explanation for the discrepancy between physical functioning factor of children and their parents is their different perception of the construction of the mentioned items. Hence, the loading of the first four items including, walking more than one block, running, doing sports activities or exercise and lifting something heavy, either in children or in their parents, were similar and were included in the same factor. All the items mentioned above are related to physical activity and were interpreted by both children and their parents in a similar manner. On the other hand, children and their parents did not have the same interpretation for the last four items including, taking a bath or shower by myself, doing chores around the house, hurting or aching and have low energy. This difference may be due to the individual perception and vision of children regarding these items, which include responsibility, independency as well as tolerability, and may explain the last two items about parents.

All items related to emotional functioning and social functioning had a clear factor loading in both self- and parent proxy-reports. The last two items of school functioning for both self- and parent proxy-report were loaded on a separate factor that was very similar to the EFA findings for the original US English version. To confirm the results of our EFA, we utilized CFA. Our results support the initial construct validity of the Iranian version of the PedsQL™ 4.0 in children aged 8-12 years.

Previous researches have presented gender differences in the pediatric HRQOL [34,40]. Our results demonstrated that girls had significantly higher HRQOL in total scale score, physical functioning, emotional functioning, social functioning and school functioning than boys, by self-report. The difference between girls and boys reflected in our data support the results of Chen et al, who used the PedsQL™ 15-item short form for adolescent girls and their parents in Japan [17]. However, our results were not consistent with our previous study and current literature [19,22,36,40]. The difference between adolescents in our previous study and children in this study may be due to the different perception of social and environmental pressure and also to puberty changes in adolescents.

The Iranian version of PedsQL™ 4.0 was able to detect the hypothesized differences between healthy and chronically ill children supporting the initial discrimination validity of the Iranian version of the PedsQL™ 4.0 for children. Consistent with other studies [8,22,41], children with chronic health conditions had lower HRQOL scores than healthy children.

Our study has several limitations. The unavailability of information on nonrespondents and investigation of only a sample from Tehran, the capital city of Iran, may limit the generalizability of the findings to other parts of Iran. Further Iranian PedsQL™ studies in the other regions in Iran are necessary. Retest reliability and responsiveness was not examined. However, it has been debated that test-retest reliability may be less useful than internal consistency reliability in HRQL instrument development [42]. Finally, the ceiling effects that been observed on some scales may limit the ability of the Iranian version of PedsQL™ 4.0 to detect HRQOL improvement in some scales for healthy children. Whether these ceiling effects are evident for chronically ill populations in Iran will require further evaluation with larger populations of ill children.

## Conclusion

Our study demonstrates the initial reliability and validity of the Iranian version of the PedsQL™ 4.0 Generic Cores Scales as an outcome measure of generic HRQOL in Iranian children aged 8-12 years old. Future research is needed with larger samples of chronically ill children aged 8-12 years, and samples of children aged 2-7 years.

## Abbreviations

**HRQOL**: Health-Related Quality of Life; **PedsQL™ 4.0**: Pediatric Quality of Life Inventory™ 4.0; **EFA**: Exploratory Factor Analysis; **CFA**: Confirmatory Factor Analysis

## Competing interests

The authors declare that they have no competing interests.

## Authors' contributions

PA designed the study, acquired, interpreted the data, drafted the manuscript, and wrote the paper. GE assisted with drafting the manuscript and dealt with the analysis process. PM assisted in data interpretation and reviewed the paper. For validation of the Iranian version of the PedsQL™ questionnaire back translation was done by NS who also critically reviewed the paper, and edited all revisions of the manuscript. MAJ contributed to the data analysis and interpretation. FA supervised and advised throughout the study. All authors read and approved the final manuscript.
